# Enriching Wheat Bread With Banana Peel Powder: Impact on Nutritional Attributes, Bioactive Compounds, and Antioxidant Activity

**DOI:** 10.1155/2024/2662967

**Published:** 2024-08-02

**Authors:** Most. Jesmin Akhter, Md. Al-Amin, Md Akram Hossain, Md. Murtuza Kamal

**Affiliations:** Department of Food Processing and Preservation Faculty of Engineering Hajee Mohammad Danesh Science and Technology University, Dinajpur 5200, Bangladesh

**Keywords:** antioxidant activity, banana peel powder, bioactive compound, bread enrichment, waste valorization

## Abstract

This research investigated the impact of enriching bread with banana peel powder (BPP) on nutritional attributes, bioactive components, antioxidant activity, and sensory characteristics. Four bread samples were prepared and evaluated: S1 (control), S2 (5% BPP), S3 (7% BPP), and S4 (10% BPP). The addition of BPP resulted in a reduction in moisture content and an increase in ash, fat, protein, and fibre levels, while reducing overall carbohydrate content. Furthermore, BPP-enriched bread exhibited an increase in total phenolic content (TPC) (ranging from 28.46 to 42.38 mg GAE/100 g) and total flavonoid content (TFC) (ranging from 6.63 to 9.46 QE mg/g), indicating enhanced antioxidant properties. The DPPH assay demonstrated the antioxidant potential of BPP-incorporated bread, with the radical scavenging activity (RSA) increasing from 18.84% to 53.03% with increasing BPP enrichment. Color assessment revealed changes in both crust and crumb, with a decrease from 78.46 to 40.53 in the lightness (*L*∗) value of the crust and from 61.21 to 41.10 in the lightness (*L*∗) value of the crumb. Additionally, changes in *a*∗ and *b*∗ values were observed. The *a*∗ values varied between 17.59 and 12.42 for the crust and between 6.96 and 5.89 for the crumb. The *b*∗ values varied between 31.61 and 23.65 for the crust and between 19.63 and 16.58 for the crumb. Sensory evaluation suggested that up to 5% BPP inclusion in bread mirrored the texture, taste, appearance, and overall acceptability of control bread, but enrichment beyond 5% resulted in lower sensory scores. In summary, the incorporation of BPP significantly influenced various aspects of bread, highlighting its potential for applications in the food and industry sectors.

## 1. Introduction

The globally cultivated banana (*Musa paradisiaca*), including plantains, is among the top 10 crops in terms of area of cultivation, yield, and calorie production [1]. It ranks as the fourth most significant crop globally, standing alongside maize, rice, and wheat [2, 3]. With an annual global production estimated at around 116 million tons [4], this fruit significantly contributes to sustaining approximately 30% of the world's population, providing both food and economic resources [2]. Additionally, bananas and plantains play a vital role in offering nutritional richness as a dietary staple across diverse regions [3].

Banana peels, constituting approximately 35%–40% of the fruit's weight, are frequently discarded as waste, accumulating nearly 36 million tons worldwide [5]. This significant disposal raises environmental concerns, as the waste releases harmful gases, namely, ammonia and hydrogen sulphide [6]. The underutilization of the massive organic mass signifies a significant economic loss [7, 8]. Banana peels are rich in dietary fibre, protein, essential amino acids, polyunsaturated fatty acids, potassium, and various antioxidant compounds such as polyphenols, carotenoids, catecholamines, and prodelphinidins [5, 9–11]. Studies suggest that banana peels contain higher mineral content and phenolic compounds, showcasing greater antioxidant activity compared to the banana pulp. Moreover, they have been utilized in various home remedies for skin problems, burns, anaemia, and other health issues [6, 12]. Due to their nutritional value, there is a considerable potential for their use in the food industry [13]. Also, the concept of transforming agrofood by-products into high-value compounds aligns with the principles of sustainability and circular economy [14].

The rising interest in reshaping waste into value, creating value-added products from organic waste, has driven an increase of research in this field. Banana peel has found applications in enhancing the fibre content of numerous food products such as meat, bread, and cookies. It serves as a source of fibre, pectin, cellulose, and pectinase [12]. Several studies have been conducted, in attempt of enhancing and fortification for different food products using the banana peel, for example, chicken sausage [15], fish patties [10], flatbread [16], biscuits [17], chapatti [18], and cookies [19].

Globally, bread holds a significant place in the diet, there is a rising trend in academic research focusing on enriching bread with a variety of dietary fibres and functional compounds. Bread, being a staple food item, is favored for its popularity and ease of preparation, making it an ideal candidate for enrichment with health-promoting components that offer increased benefits to consumers [20–22]. Bread is mostly produced from wheat flour, but the bread production is seeing a surge in the utilization of composite flours in response to the growing demand for functional foods, which provide advantageous effects against various diseases, as well as to mitigate wheat import dependencies in many countries [23–25]. Banana Peel Powder (BPP) is rich in fibre, protein, antioxidants, and other nutritional components. By enriching bread with BPP, it can be transformed into a functional food, enhancing its nutritional and functional properties.

Therefore, the current research is aimed at determining the proximate composition, bioactive compounds, antioxidative activity, and sensory attributes of wheat bread enriched with BPP. It is hypothesized that BPP enrichment will enhance the nutritional and bioactive properties, as well as the antioxidant capacity of the bread.

## 2. Materials and Methods

This study was conducted in the Food Processing and Preservation Laboratory of Hajee Mohammad Danesh Science and Technology University, Bangladesh. Fully ripe and undamaged local malbhog (*Musa paradisiaca* AAB group) variety bananas and other ingredients were procured from the local market. All required analytical grade chemicals were used from laboratory stock.

### 2.1. Preparation of BPP

Following the process detailed by Kabir et al. [26], the procured bananas were washed using both chlorinated and distilled water, followed by the manual peeling. To prevent browning, the peels were dipped in a solution (0.5% w/v citric acid and 0.1% Potassium Metabisulphite) for 20 min. After draining, the peels were thinly sliced and subsequently dried in a cabinet dryer at 60 ± 5°C. After drying, the dried peels were made into powder using a grinder machine (Jaipan JFM 1300). The powders were then sieved through sieve no. 80, to get particle size below 0.18 mm. The sieved powders were packed into airtight low-density polyethylene bags and stored in a cool place for further physicochemical analysis and preparation of enriched bread.

### 2.2. Preparation of Breads

The straight-dough method was used to make bread, a slightly modifying method detailed by Chaple et al. [27]. The method involved using 100 g wheat flour, 3.5 g instant active dry yeast, 0.4 g salt, 16.0 g sugar, 10.0 g oil, and 60 ml water for control bread preparation. Flour was substituted by different percentages of BPP (Table 1) for preparing enriched bread samples.

**Figure 1:**
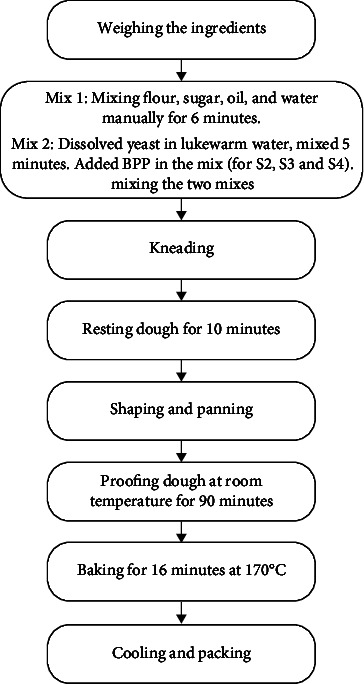
Flowchart for preparation of bread samples.

The modified method of bread preparation is outlined in Figure 1.

### 2.3. Proximate Analysis

The proximate compositions, including moisture, ash, fat, protein, and crude fibre in the samples, were assessed using AOAC methods with slight modifications as outlined by Hossain et al. [28, 29]. These methods involved oven drying, a muffle furnace, Soxhlet extraction, and the Kjeldahl apparatus for determining moisture, ash, fat, and protein, respectively. The acid–alkaline digestion method was employed for crude fibre analysis. Additionally, the carbohydrate content of the samples was calculated by subtracting the values of moisture, ash, fat, fibre, and protein from 100 [30].

### 2.4. Determination of Bioactive Compounds

#### 2.4.1. Organic Solvent Extraction for the Analysis of Bioactive Compounds

Following the procedure detailed by Islam et al. [31], BPP and BPP-enriched bread samples underwent extraction using methanol. Each 2.5 g sample was mixed with 50 mL of 80% methanol in a conical flask, maintaining a solid/liquid ratio of 1 : 20 (g/mL) for the extraction of bioactive compounds. The extraction process occurred at room temperature, with stirring at 100 rpm in a water bath for 60 min. Following this, the sample underwent centrifugation for 10 min at 4000 rpm using a standard centrifuge (MF-300, Human Lab Instrument Co., Korea). Subsequently, the supernatant was extracted using a 10 mL plastic syringe and filtered through Whatman no. 1 filter paper. The clarified supernatant was then transferred for analysis.

#### 2.4.2. Determination of Total Phenolic Content (TPC)

The TPC was evaluated through the Folin–Ciocalteu assay method detailed by Hasan et al. [32] with slight modifications. A 10 mL solution was prepared by combining 0.5 mL of sample extract, 0.5 mL of Folin–Ciocalteu solutions, and 1 mL of sodium bicarbonate (7.5% solution) and adjusting the volume with distilled water. Following brief vortexing, the solutions were left at room temperature for 35 min in a dark area and then centrifuged for 10 min at 4000 rpm. Using a UV-VIS spectrophotometer (UV 1900i, Shimadzu, Japan), the absorbance was measured at 750 nm, with background subtraction using a suitable blank. The outcomes are presented as milligrams of gallic acid equivalent per 100 gram of dry matter (mg GAE/100 g DM), calibrated against a standard curve using gallic acid.

#### 2.4.3. Determination of Total Flavonoid Content (TFC)

The TFC was determined utilizing the colorimetric method as outlined by Rahman, de Camargo, and Shahidi [33] with minor adjustments. Approximately 1 mL of extract was combined with 4 mL of distilled water and 0.3 mL of 5% NaNO_2_ solution in 15 mL falcon tubes. After standing for 5 min, 0.3 mL of 10% AlCl_3_ was added and left for another 1 min. Then, 2.4 mL of distilled water and 2 mL of 1 M NaOH were added to the tubes and thoroughly mixed. The falcon tubes with the mixture were centrifuged for 10 min at 4000 rpm. The centrifuged tubes were allowed to incubate for 15 min in a dark room. After that, the absorbance of the supernatant was measured at 510 nm. A blank was also prepared using a similar method but with methanol instead of the sample extract. The total flavonoid concentration was quantified using quercetin's standard curve and expressed as mg of quercetin equivalent per gram of dry matter (mg QE/g DM).

#### 2.4.4. Determination of Radical Scavenging Activity (RSA) by DPPH Assay

The RSA of the samples was evaluated following the method detailed by Zhang et al. [34]. Initially, a 0.1 mM DPPH solution was prepared using 80% (V/V) methanol. Subsequently, 50 *μ*L of the extracted sample was mixed with 1.95 mL of the 0.1 mM DPPH solution and thoroughly vortexed. A control sample of 2 mL of DPPH solution without any sample was also prepared. The mixtures were then left in a dark place for 30 min. Afterward, their absorbance at 515 nm was measured using a spectrophotometer. The RSA (%) was calculated using Equation (1).

(1) $$\begin{array}{c} \text{RSA }\left(\%\right)=\frac{A_{\text{Control}}-A_{\text{Sample}}}{A_{\text{Control}}}\times 100 \end{array}$$

where *A* represents the absorbance at 515 nm.

### 2.5. Color Evaluation of Bread

The color evaluation of both the bread crust and crumb was conducted utilizing a handheld colorimeter (BCM-200, Biobase, China). The assessment involved determining the *L*∗, *a*∗, and *b*∗ values. Calibration was performed using a standard white tile. The *a*∗ value signifies greenness or redness, and the *b*∗ value indicates yellowness or blueness, with both values ranging from -120 to +120. The *L*∗ value measures brightness or lightness, ranging from zero (black) to 100 (white).

### 2.6. Sensory Evaluation

A panel of 30 (18 males and 12 females) semitrained panellists, age ranging from 20–36 years, evaluated the sensory characteristics of the prepared bread using a 9-point hedonic rating system, considering attributes such as color, texture, flavor, and overall appeal of the crust (9-*liked extremely*, 8-*liked very much*, 7-*liked moderately*, 6-*liked slightly*, 5-*neither liked nor disliked*, 4-*disliked slightly*, 3-*disliked moderately*, 2-*disliked very much*, and 1-*disliked extremely*). To minimize bias, each judge received bread samples (one 30 gm loaf in separate trays) labelled with distinct random numbers, along with water to cleanse their palates before and during the evaluation.

### 2.7. Statistical Analysis

Each experiment was replicated three times. The collected data was analyzed using SPSS (version 25.0). The results were reported as mean and standard deviation. To assess significant differences between the groups, one-way analysis of variance (ANOVA) was performed, followed by Duncan's multiple range test (DMRT) with a confidence level of 95% to differentiate between the means.

## 3. Results and Discussion

### 3.1. Proximate Composition

The results of the proximate analysis of the BPP are depicted in Table 2. In terms of moisture content, BPP exhibited a moisture level of 7.05%, comparable to a study by Eshak [16] on BPP, which found 6.39% moisture. The slight variation in moisture content could be attributed to differences in drying environment and method employed in the respective studies.

Moving on to the moisture content of the enriched bread samples, it ranged between 24.01% and 27.00% (Table 3). Among the formulated bread, S4 showed the lowest moisture content at 24.01%, while the control (S1) had the highest at 27.00%. The control bread in this study had more moisture content than that found by Upadhyay et al. [35] at 20.31%. Conversely, Begum, Chakraborty, and Deka [36], in a study utilizing banana bract for bread enrichment, observed higher moisture levels ranging from 30.28% to 35.27%. Also, the standard initial moisture content of the bread can be as high as 35.00% to 40.00% [37]. It is worth noting that variations in the initial moisture content of the wheat flour used could contribute to these discrepancies. Additionally, as BPP enrichment increased, a corresponding reduction in moisture content was observed. This can be attributed to the inherently lower moisture content of BPP compared to wheat flour, resulting in a dilution effect as BPP concentration increased within the formulations. Segura-Badilla et al. [38] also reported a similar increase due to addition of BPP to bread.

BPP exhibited an ash content of 12.19% (Table 2), consistent with the findings of Zubair, Esrafil, and Kona [39] and Lee et al. [40] who reported approximately 11.47% and 13.22%, respectively. The control bread, without BPP, demonstrated an ash content of 0.92% (Table 3), aligning closely with the 1.20% reported by Aly et al. [41]. With increasing percentages of BPP added, the ash content of the prepared bread also exhibited a clear upward trend, ranging from 1.23% to 2.03% (Table 3). This rise in ash content correlates with the substantial disparity between the ash content of wheat flour, approximately 1.61% as reported by Dhillon, Choudhary, and Sodhi [42], and that of BPP, which stands at 12.19%. The increase in ash content suggests the presence of minerals contributed by BPP in the enriched breads. Therefore, the incorporation of BPP into the bread formulation inevitably leads to increases in the overall ash content.

In a study by Mohd Zaini et al. [43], the fat content of BPP was found to be 4.08% for BPP of the Berangan variety. Our prepared BPP exhibited a comparable fat content of 3.92%, aligning closely with their findings. Similarly, the fat content of the control bread sample in our study, at 2.89%, compares well with the 2.48% reported by Rahman et al. [44]. As BPP enrichment increased, the fat content in the bread samples also demonstrated a statistically significant increase, ranging from 4.38% to 5.92% (Table 3). This observed trend is logical, as BPP contains 3.92% fat, whereas wheat flour, as reported by Awol et al. [45], has a lower fat content ranging from 1.25% to 0.39%. Additionally, banana peel, as noted by Alam et al. [46], possesses an oil holding capacity of 0.44 g oil/g dry sample, further clarifying the increase in fat content with the increase of banana peel enrichment. Oil holding capacity of bread also increases with the increase in BPP enrichment as highlighted by Eshak [16]. Similar phenomena of increasing fat content of the enriched products were also observed by Segura-Badilla et al. [38] while enriching pasta, bread, and biscuits using BPP.

The protein content in BPP was determined to be 9.29% (Table 2), closely resembling the 9.30% reported by Singh et al. [47] and 9.66% reported by Zubair, Esrafil, and Kona [39]. In contrast, the control enriched bread sample in our study exhibited a protein content of 9.14% (Table 3), higher than the 7.61% reported by Belc et al. [48] and lower than the 13.83% observed in the control sample by Johnston et al. [49]. Wheat flour serves as the main protein source in bread, with protein content influenced by wheat variety. The slight disparities observed between our study and others could be attributed to differences in wheat varieties and the protein content of the wheat flour used in bread making. As the enrichment of BPP increased in the formulated bread samples, protein content also rose, ranging from 10.73% to 11.93%. This trend suggests that BPP, with a higher protein content compared to wheat flour (9.29% in our case), contributed to the increased protein content of the enriched bread. Similar findings were reported by Segura-Badilla et al. [38], who observed a slight increase in protein content when using 10% BPP for bread enrichment compared to the control sample.

The fibre content in BPP was measured at 11.70% (Table 2), slightly lower than the 16.66% reported by Akram et al. [9] in another variety of banana (*Musa balbisiana*), but similar to the results from Eshak [16] of 11.20% and Zubair, Esrafil, and Kona [39] of 12.43% fibre content. In our study, the fibre content of enriched bread samples exhibited a statistically significant increase with increasing BPP enrichment, ranging from 1.98% to 2.82% (Table 3), whereas the control sample had only 0.25% fibre content. This significant increase can be attributed to the higher fibre content of BPP (11.70% in our case) compared to wheat flour (0.34% as reported by Alam et al. [46]). Similar findings were observed in studies by Segura-Badilla et al. [38] and Alam et al. [46], where bread and cookies enriched with BPP showed increased fibre content with higher BPP enrichment levels.

The carbohydrate content of BPP was measured at 67.53% (Table 2), closely resembling findings of 78.72% by Singh et al. [47] and 61.45% by Akram et al. [9]. In our study, as BPP enrichment increased, a negative trend in carbohydrate content was observed in the bread samples ranging from 64.04% to 56.11% (Table 3). This trend can be attributed to the fact that wheat flour, the main component of bread, typically contains more carbohydrate than BPP. As BPP replaced wheat flour and contributed less carbohydrate, the total carbohydrate content of the bread decreased. Mohd Zaini et al. [12] reported that banana peel may have carbohydrate content ranging from 59.51% to 76.58%, while Memon et al. [50] found that wheat flour has carbohydrate content ranging from 78.70% to 81.90%. This suggests that the substitution of wheat flour with BPP influences the carbohydrate composition of the bread samples.

### 3.2. Bioactive Compounds

#### 3.2.1. TPC

Phenolic compounds are known for their antioxidant properties and potential health benefits. In our study, the TPC in BPP was measured at 162.52 mg GAE/100 g (Table 4), which, although lower than the findings of Rita et al. [51] at 273.09 mg GAE/100 g, still underscores the significant presence of phenolic compounds in banana peel. The variation in phenolic content between studies may be attributed to factors such as fruit maturity, variety, and analytical techniques. For instance, the decrease in phenolic content with the ripening of bananas, as reported by Vu, Scarlett, and Vuong [52], could explain the lower phenolic content observed in our study, as we utilized the peels of ripened bananas.

**Figure 2:**
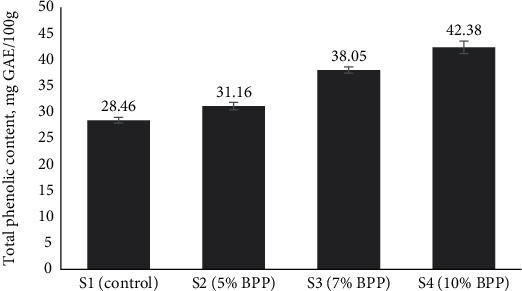
Total phenolic content (TPC) of BPP-enriched bread samples. Here, BPP = banana peel powder, S1 = control bread (0% BPP), S2 = 5% BPP-incorporated bread, S3 = 7% BPP-incorporated bread, S4 = 10% BPP-incorporated bread.

Additionally, Zhang et al. [53] highlighted the variability in TPC of wheat flour across different varieties and storage periods, further emphasizing the influence of such factors on research outcomes. In our investigation, the substitution of wheat flour with freshly prepared BPP led to a notable increase in the TPC of the enriched bread samples. As BPP enrichment increased, the TPC in the bread samples exhibited an upward trend, ranging from 28.46 to 42.38 mg GAE/100 g (Figure 2). Similar increases upon enrichment of bread were noted in other studies by Ertosun et al. [54] and Oyinloye et al. [55]. This trend suggests that the enrichment of bread with BPP could potentially enhance its antioxidant properties and offer health benefits to consumers seeking antioxidant-rich food options.

#### 3.2.2. TFC

Flavonoids are well-known for their antioxidant and anti-inflammatory properties, which contribute to their potential health benefits. The TFC in BPP measured at 19.89 QE mg/g (Table 4) signifies the presence of these bioactive compounds in banana peel. This finding is markedly higher than the TFC of BPP reported by Zubair, Esrafil, and Kona [39] at 6.15 QE mg/g, highlighting the considerable variability in flavonoid content among different BPP samples, likely influenced by factors such as banana variety and processing methods.

**Figure 3:**
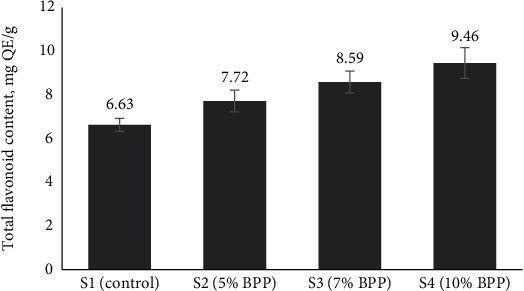
Total flavonoid content (TFC) of BPP-enriched bread samples. Here, BPP = banana peel powder, S1 = control bread (0% BPP), S2 = 5% BPP-incorporated bread, S3 = 7% BPP-incorporated bread, S4 = 10% BPP-incorporated bread.

It is noteworthy that flavonoid content can also be influenced by storage time, as reported by Zhang et al. [53]. We hypothesize that the flavonoid content of BPP was higher than that of wheat flour at the time of use. So, as the BPP enrichment increased, the TFC in the bread samples also exhibited an increase, ranging from 6.63 to 9.46 QE mg/g (Figure 3). This trend suggests that the enrichment of bread with BPP could effectively enhance the flavonoid content, thereby potentially improving the health-promoting properties of the enriched bread. Consistent with our findings, similar increases in flavonoid content upon enrichment have been reported in studies by Ateeq et al. [56] and Dossa et al. [57], highlighting the robustness of our results and the potential of BPP as a good source of flavonoids for functional food applications.

#### 3.2.3. RSA by DPPH Assay

The RSA of BPP, measured using the DPPH assay, was found to be 66.12% (Table 4), highlighting its significant antioxidant potential in combating oxidative stress and protecting against cell damage. This finding is consistent with previous studies by Toupal and Coşansu [58] and Aboul-Enein et al. [59], who reported scavenging activity levels of 50.03% and a range of 40.45%–46.63%, respectively, for banana peel.

**Figure 4:**
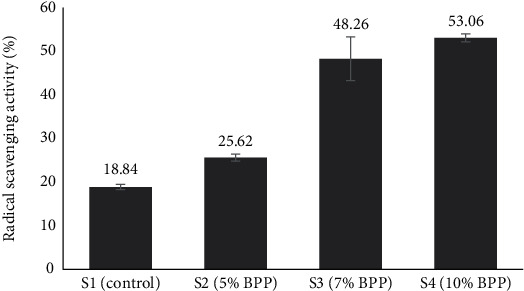
Radical scavenging activity (%) of BPP-enriched bread samples. Here, BPP = banana peel powder, S1 = control bread (0% BPP), S2 = 5% BPP-incorporated bread, S3 = 7% BPP-incorporated bread, S4 = 10% BPP-incorporated bread.

The control bread sample exhibited RSA of 18.84%, consistent with the findings by Ragaee et al. [60], serving as a baseline for comparison. In contrast, the enriched bread samples demonstrated a range of scavenging activity from 25.62% to 53.03%, with higher levels observed in samples with greater BPP enrichment (Figure 4). While these levels were lower than those reported by Olugbuyi et al. [24] for bread enriched with green plantain-amaranth, ranging from 51.35% to 69.76%, they still suggest the potential of BPP-enriched bread to balance reactive oxygen species and mitigate oxidative stress [61]. The observed scavenging activity levels highlight the importance of incorporating BPP into bread formulations as a means of enhancing their antioxidant properties thus promoting consumer health.

### 3.3. Color Evaluation of Bread

Color is a vital aspect of consumer acceptance in baked goods, including bread, influencing the overall perception and appeal of the product [62]. The color evaluation presented in Table 5 reveals the significant impact of BPP on the color characteristics of both the crust and crumb in the prepared bread samples.

In terms of crust color, the *L*∗ values decrease with higher percentages of BPP incorporation, indicating darker crusts compared to the control sample (S1) with 0% BPP. Samples enriched with BPP (S2, S3, and S4) display lower *L*∗ values, ranging from 39.66 to 46.12, indicative of darker crusts. Additionally, variations in the *a*∗ and *b*∗ values, representing color tone and saturation, reveal distinct color characteristics among the BPP-enriched samples. While the control sample (S1) exhibits a reddish hue with moderate yellowness, samples with BPP enrichment (particularly S2 and S4) display slightly redder hues and reduced yellowness. These observations suggest that BPP incorporation influences both the tone and saturation of the crust color.

Turning to the crumb color, the analysis reveals significant changes attributable to BPP incorporation. The *L*∗ values, indicating lightness, decrease with higher BPP content, resulting in darker crumbs (Table 5). Specifically, the control sample (S1) exhibits an *L*∗ value of 61.21, while samples with BPP enrichment (S2, S3, and S4) show *L*∗ values ranging from 41.10 to 52.05, indicating darker crumbs. Similarly, the *a*∗ values, representing redness, exhibit a slight decrease with BPP enrichment, while the *b*∗ values, indicating yellowness, show a slight increase. These findings suggest that BPP incorporation leads to a darker and less yellow appearance in the crumb of the bread samples.

The observed changes in crust and crumb color are likely attributed to the presence of phenolic compounds, carotenoids, and xanthophylls in banana peel, along with reactions such as the Maillard reaction, caramelization, and enzymatic browning during baking [18, 63]. These findings align with previous observations by Ayoub et al. [64] during the preparation of biscuits using BPP and other fibre-rich components. However, further research is needed to fully understand the underlying mechanisms driving these color changes and to optimize color parameters to meet consumer preferences and ensure product quality in the development of functional bakery products enriched with BPP.

### 3.4. Sensory Evaluation of Bread

**Figure 5:**
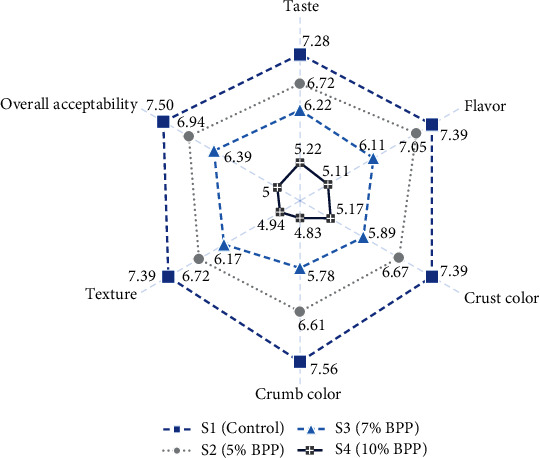
Sensory evaluation scores of control and BPP-enriched bread. Here, BPP = banana peel powder, S1 = control bread (0% BPP), S2 = 5% BPP-incorporated bread, S3 = 7% BPP-incorporated bread, S4 = 10% BPP-incorporated bread.

The sensory analysis depicted in Figure 5 of the bread samples reveals a noticeable impact of BPP enrichment on consumer perceptions. As discussed earlier regarding color evaluation, the presence of phenolic and other bioactive components in BPP has influenced the color of both the crust and crumb, resulting in slightly darker bread, which consequently scored lower compared to the control sample. Besides the color of the bread, the increase in BPP in bread is also affecting taste, flavor, and texture as perceived by the panellists. The astringency and slight bitterness of the BPP, attributed to the flavonoid phenols present in the peel, may explain the lower scores in taste and flavor. Additionally, the chewy texture of bread, largely dependent on the gluten in wheat flour, is compromised when substituted with gluten-free BPP, resulting in softer texture in the enriched bread samples, which also scored comparatively lower. The overall acceptability of the bread indicates that the sample enriched with 5% BPP (S2) closely mirrors the scores of the control bread and is most favored among the enriched samples, emphasizing the potential of 5% BPP enrichment to maintain sensory appeal while obtaining nutritional benefits. Such changes in sensory attributes due to addition of BPP have been reported by other studies as well, during preparation of flatbread [16, 18], bread [65], biscuits [64, 66], and cookies [67]. While the study highlights the positive nutritional aspects of the enriched bread over the control bread, it also underscores the challenges in meeting sensory expectations. This suggests the necessity for a balanced approach in formulating enriched bread, considering both nutritional benefits and consumer preferences.

## 4. Conclusion

The research is aimed at assessing the proximate composition, bioactive compounds, antioxidative activity, and sensory attributes of wheat bread enriched with BPP. Our hypothesis suggested that BPP enrichment would enhance the nutritional profile and bioactive properties of bread. The findings indicate significant improvements in the nutritional and bioactive aspects of bread with BPP incorporation. Enriched bread samples exhibited elevated levels of protein, fat, ash, and fibre, while reducing carbohydrate content compared to the control wheat flour bread. Additionally, BPP-enriched bread displayed enhanced antioxidant activity, demonstrated by increased total phenolic and flavonoid content, along with improved RSA. Sensory evaluation revealed that up to 5% BPP enrichment maintained overall acceptability comparable to the control bread. However, higher BPP concentrations resulted in decreased sensory scores. These findings highlight the potential of BPP for applications in the food industry, offering a means to enhance both the nutritional profile and antioxidant activity of bread products. Further research could explore optimal enrichment levels of BPP to balance nutritional benefits with sensory attributes of other products, ensuring consumer acceptance while maximizing health-promoting properties.

## Figures and Tables

**Table 1:** Percent BPP used in bread formulation.

**Sample name**	**BPP (%)**
S1 (control)	0
S2	5
S3	7
S4	10

**Table 2:** Proximate composition (%) of banana peel powder (BPP).

**Sample**	**Moisture**	**Ash**	**Fat**	**Protein**	**Fibre**	**Carbohydrate**
BPP	7.05 ± 0.08	12.19 ± 0.04	3.92 ± 0.09	9.29 ± 0.16	11.70 ± 0.20	67.53 ± 0.13

*Note:* Values are expressed as means ± standard deviation.

**Table 3:** Proximate composition (%) of BPP-enriched bread samples.

**Sample**	**Moisture**	**Ash**	**Fat**	**Protein**	**Fibre**	**Carbohydrate**
S1 (0% BPP)	27.00 ± 1.03^a^	0.92 ± 0.03^a^	2.89 ± 0.03^a^	9.14 ± 0.17^a^	0.25 ± 0.02^a^	64.04 ± 0.81^a^
S2 (5% BPP)	25.72 ± 0.86^ab^	1.23 ± 0.03^b^	4.38 ± 0.06^b^	10.73 ± 0.25^b^	1.98 ± 0.03^b^	57.93 ± 1.03^b^
S3 (7% BPP)	24.65 ± 0.83^ab^	1.79 ± 0.02^c^	5.18 ± 0.05^c^	11.18 ± 0.06^c^	2.22 ± 0.10^b^	57.20 ± 0.87^b^
S4 (10% BPP)	24.01 ± 2.22^b^	2.03 ± 0.03^d^	5.92 ± 0.05^d^	11.93 ± 0.04^d^	2.82 ± 0.06^c^	56.11 ± 2.15^b^

*Note:* Values are expressed as means ± standard deviation. Different superscript letters within a column signify significant differences (*p* ≤ 0.05).

Abbreviations: BPP = banana peel powder, S1 = control bread (0% BPP), S2 = 5% BPP-incorporated bread, S3 = 7% BPP-incorporated bread, S4 = 10% BPP-incorporated bread.

**Table 4:** Bioactive compounds of BPP.

**Sample**	**TPC (mg GAE/100 g)**	**TFC (QE mg/g)**	**RSA (%)**
BPP	162.52 ± 0.24	19.89 ± 0.08	66.12 ± 0.15

*Note:* Values are expressed as means ± standard deviation.

**Table 5:** Color values of control and BPP-enriched bread.

		**L**∗	**a**∗	**b**∗
Crust	S1 (0% BPP)	78.46 ± 0.14^e^	15.18 ± 0.09^d^	31.61 ± 0.10^e^
	S2 (5% BPP)	39.66 ± 0.11^a^	17.59 ± 0.13^e^	24.20 ± 0.08^c^
	S3 (7% BPP)	46.12 ± 0.10^b^	12.42 ± 0.12^c^	26.71 ± 0.12^d^
	S4 (10% BPP)	40.53 ± 0.16^a^	15.20 ± 0.10^d^	23.65 ± 0.11^c^

Crumb	S1 (0% BPP)	61.21 ± 0.12^d^	6.96 ± 0.11^a^	16.58 ± 0.08^a^
	S2 (5% BPP)	52.05 ± 0.10^c^	6.42 ± 0.08^b^	18.86 ± 0.12^b^
	S3 (7% BPP)	49.93 ± 0.13^bc^	6.49 ± 0.12^b^	19.63 ± 0.09^b^
	S4 (10% BPP)	41.10 ± 0.15^a^	5.89 ± 0.12^b^	19.12 ± 0.14^b^

*Note:* Values are expressed as means ± standard deviation. Different superscript letters within a column signify significant differences (*p* ≤ 0.05).

Abbreviations: *a*∗ = green to red axis (−a to +a), *b*∗ = blue to yellow axis (−b to +b), BPP = banana peel powder, *L*∗ = level of lightness, S1 = control bread (0% BPP), S2 = 5% BPP-incorporated bread, S3 = 7% BPP-incorporated bread, S4 = 10% BPP-incorporated bread.

## Data Availability

All the data used in this study is included in the manuscript itself.

## References

[1] Varma V., Bebber D. P. (2019). Climate change impacts on banana yields around the world. *Nature Climate Change*.

[2] Lucas S. S. (2021). The status of banana production in Tanzania; a review of threats and opportunities. *International Journal of Current Science Research and Review*.

[3] Patrick S., Mirau S., Mbalawata I., Leo J. (2023). Time series and ensemble models to forecast banana crop yield in Tanzania, considering the effects of climate change. *Resources, Environment and Sustainability*.

[4] Srivastava P. N., Kumar D., Raghavendra S., Alapati P., Shamim M. D., Deepti (2023). Tissue culture technology intervention in Red Dacca and Cavendish banana for nutritive value enhancement under organic farming in India. *In Transforming Organic Agri-Produce into Processed Food Products*.

[5] Gomes S., Vieira B., Barbosa C., Pinheiro R. (2022). Evaluation of mature banana peel flour on physical, chemical, and texture properties of a gluten‐free Rissol. *Journal of Food Processing and Preservation*.

[6] Kamel N. A., El-messieh S. L. A., Saleh N. M. (2017). Chitosan/banana peel powder nanocomposites for wound dressing application: preparation and characterization. *Materials Science and Engineering: C*.

[7] Bashmil Y. M., Ali A., Bk A., Dunshea F. R., Suleria H. A. R. (2021). Screening and characterization of phenolic compounds from Australian grown bananas and their antioxidant capacity. *Antioxidants*.

[8] Zaini H. M., Saallah S., Roslan J. (2023). Banana biomass waste: a prospective nanocellulose source and its potential application in food industry – a review. *Heliyon*.

[9] Akram T., Mustafa S., Ilyas K. (2022). Supplementation of banana peel powder for the development of functional broiler nuggets. *Peer J*.

[10] Bin Mohd Zaini H., Bin Sintang M. D., Dan Y. N., Ab Wahab N., Bin Abdul Hamid M., Pindi W. (2019). Effect of addition of banana peel powder (Musa balbisiana) on physicochemical and sensory properties of fish patty. *British Food Journal*.

[11] Ijaz N., Afzaal M., Niaz B. (2023). Structural and functional investigations of wall material extracted from banana peels. *International Journal of Food Properties*.

[12] Mohd Zaini H., Roslan J., Saallah S., Munsu E., Sulaiman N. S., Pindi W. (2022). Banana peels as a bioactive ingredient and its potential application in the food industry. *Journal of Functional Foods*.

[13] Kumari P., Gaur S. S., Tiwari R. K. (2023). Banana and its by-products: a comprehensive review on its nutritional composition and pharmacological benefits. *eFood*.

[14] Pinela J., Carvalho A. M., Ferreira I. C. (2017). Wild edible plants: nutritional and toxicological characteristics, retrieval strategies and importance for today’s society. *Food and Chemical Toxicology*.

[15] Zaini H. B. M., Sintang M. D. B., Pindi W. (2020). The roles of banana peel powders to alter technological functionality, sensory and nutritional quality of chicken sausage. *Food Science & Nutrition*.

[16] Eshak N. S. (2016). Sensory evaluation and nutritional value of balady flat bread supplemented with banana peels as a natural source of dietary fiber. *Annals of Agricultural Sciences*.

[17] Yasin N. H., Ibrahim M. B., Khan N. F., Awang Z. B., Halim M. H., Saidin S. S., Mansour N., Bujosa Vadell L. M. (2023). From waste to health: an innovation of high-fiber biscuit using Brown Rice and Banana Peel. *Finance, Accounting and Law in the Digital Age: the Impact of Technology and Innovation in the Financial Services Sector*.

[18] Kurhade A., Patil S., Sonawane S. K., Waghmare J. S., Arya S. S. (2016). Effect of banana peel powder on bioactive constituents and microstructural quality of chapatti: unleavened Indian flat bread. *Journal of Food Measurement and Characterization*.

[19] Arun K., Persia F., Aswathy P. (2015). Plantain peel - a potential source of antioxidant dietary fibre for developing functional cookies. *Journal of Food Science and Technology*.

[20] De Lamo B., Gómez M. (2018). Bread enrichment with oilseeds. A review. *Foods*.

[21] Betoret E., Rosell C. M. (2020). Enrichment of bread with fruits and vegetables: Trends and strategies to increase functionality. *Cereal Chemistry*.

[22] Khoozani A. A., Kebede B., Bekhit A. E.-D. A. (2020). Rheological, textural and structural changes in dough and bread partially substituted with whole green banana flour. *Lwt*.

[23] Holmes J. T., Jaberansari Z., Collins W., Latour M. L., Modulevsky D. J., Pelling A. E. (2022). Homemade bread: repurposing an ancient technology for in vitro tissue engineering. *Biomaterials*.

[24] Olugbuyi A. O., Adepeju A. B., Ayodele B. O., Oluwajuyitan T. D. (2023). Mature green plantain-amaranth flour inclusion improved wheat bread nutrients, antioxidant activities, glycemic index/load and carbohydrate hydrolyzing enzyme inhibitory activities. *Food Chemistry Advances*.

[25] del Carmen Robles-Ramírez M., Ortega-Robles E., Monterrubio-López R., Mora-Escobedo R., del Carmen Beltrán-Orozco M. (2020). Barley bread with improved sensory and antioxidant properties. *International Journal of Gastronomy and Food Science*.

[26] Kabir M. R., Hasan M. M., Islam M. R., Haque A. R., Hasan S. M. K. (2021). Formulation of yogurt with banana peel extracts to enhance storability and bioactive properties. *Journal of Food Processing and Preservation*.

[27] Chaple S., Sarangapani C., Dickson S., Bourke P. (2023). Product development and X-ray microtomography of a traditional white pan bread from plasma functionalized flour. *LWT*.

[28] Hossain M. A., Dewan M. F., Billah M. T., Ahiduzzaman M., Haque M. M., Haque M. A. (2023). Jackfruit seed as a natural source for protein and mineral enrichment of yogurt. *Journal of Food Processing and Preservation*.

[29] AOAC (2005). *Official Methods of Analysis*.

[30] FAO (2003). *Food Energy: Methods of Analysis and Conversion Factors: Report of a Technical Workshop, Rome, 3-6 December, 2002*.

[31] Islam M. R., Haque A. R., Kabir M. R., Hasan M. M., Khushe K. J., Hasan S. K. (2021). Fruit by-products: the potential natural sources of antioxidants and *α*-glucosidase inhibitors. *Journal of Food Science and Technology*.

[32] Hasan S. M. K., Asaduzzaman M., Merkyte V., Morozova K., Scampicchio M. (2018). Simultaneous kinetic and thermodynamic-based assay to determine the hydrogen peroxide (H2O2) scavenging activity of berry extracts by using reaction calorimetry. *Food Analytical Methods*.

[33] Rahman M. J., de Camargo A. C., Shahidi F. (2017). Phenolic and polyphenolic profiles of chia seeds and their in vitro biological activities. *Journal of Functional Foods*.

[34] Zhang L., Tu Z., Yuan T., Wang H., Xie X., Fu Z. (2016). Antioxidants and *α*-glucosidase inhibitors from Ipomoea batatas leaves identified by bioassay-guided approach and structure-activity relationships. *Food Chemistry*.

[35] Upadhyay S., Tiwari R., Kumar S. (2023). Utilization of food waste for the development of composite bread. *Sustainability*.

[36] Begum Y. A., Chakraborty S., Deka S. C. (2020). Bread fortified with dietary fibre extracted from culinary banana bract: its quality attributes and in vitro starch digestibility. *International Journal of Food Science & Technology*.

[37] Rammal M., Badran A., Haidar C. (2024). Cymbopogon winterianus (Java citronella plant): a multi-faceted approach for food preservation, insecticidal effects, and bread application. *Foods*.

[38] Segura-Badilla O., Kammar-García A., Mosso-Vázquez J. (2022). Potential use of banana peel (*Musa cavendish*) as ingredient for pasta and bakery products. *Heliyon*.

[39] Zubair M. A., Esrafil M., Kona F. T. (2023). Estimation of nutritional composition of kitchen wastes and comparison of the effect of different drying methods on bioactive compounds in the wastes. *Food and Humanity*.

[40] Lee E.-H., Yeom H.-J., Ha M.-S., Bae D.-H. (2010). Development of banana peel jelly and its antioxidant and textural properties. *Food Science and Biotechnology*.

[41] Aly A. A., Abusharha A., El-Deeb F. E., Abdelazeem A. A. (2023). Effects of adding whole barley flour to bread and its impact on anti-obesity action of female rats fed a high-fat diet. *Arabian Journal of Chemistry*.

[42] Dhillon B., Choudhary G., Sodhi N. S. (2020). A study on physicochemical, antioxidant and microbial properties of germinated wheat flour and its utilization in breads. *Journal of Food Science and Technology*.

[43] Mohd Zaini H., Sintang M. D. B., Roslan J. (2021). Functional and sensorial properties of chicken sausage supplemented with banana peel flours of different varieties. *Applied Sciences*.

[44] Rahman T., Akter S., Sabuz A. A., Rana R. (2021). Characterization of wheat flour bread fortified with banana flour. *International Journal of Food Science and Agriculture*.

[45] Awol S. M., Kuyu C. G., Bereka T. Y., Abamecha N. (2024). Physicochemical stability, microbial growth and sensory quality of refined wheat flour as affected by packaging materials during storage. *Journal of Stored Products Research*.

[46] Alam M. J., Akter S., Afroze S., Islam M. T., Sayeem E. H. (2020). Development of fiber and mineral enriched cookies by utilization of banana and banana peel flour. *Journal of Microbiology, Biotechnology and Food Sciences*.

[47] Singh R., Deshpande A. S., Pallavi A., Kamboj V. (2023). Sustainable use of waste banana peel (*Musa* × *sapientum* L.) powder for enhancement of nutritional properties of dark chocolate. *AgroEnvironmental Sustainability*.

[48] Belc N., Duta D. E., Culetu A., Stamatie G. D. (2021). Type and amount of legume protein concentrate influencing the technological, nutritional, and sensorial properties of wheat bread. *Applied Sciences*.

[49] Johnston C., Leong S. Y., Teape C., Liesaputra V., Oey I. (2023). In vitro digestion properties and use of automatic image analysis to assess the quality of wheat bread enriched with whole faba bean (*Vicia faba* L.) flour and its protein-rich fraction. *Food Research International*.

[50] Memon A. A., Mahar I., Memon R. (2020). Impact of flour particle size on nutrient and phenolic acid composition of commercial wheat varieties. *Journal of Food Composition and Analysis*.

[51] Rita W., Swantara I., Asih I., Damayanti N. (2023). Antibacterial efficacy of susu banana (*Musa paradisiaca* L.) peel methanol extract and the total contents of flavonoid and phenolic compounds. *IOP Conference Series: Earth and Environmental Science*.

[52] Vu H. T., Scarlett C. J., Vuong Q. V. (2019). Changes of phytochemicals and antioxidant capacity of banana peel during the ripening process; with and without ethylene treatment. *Scientia Horticulturae*.

[53] Zhang Y., Truzzi F., D’Amen E., Dinelli G. (2021). Effect of storage conditions and time on the polyphenol content of wheat flours. *Processes*.

[54] Ertosun S., Falcão S. I., Aylanc V. (2024). The impact of bee product incorporation on the processing properties, nutritional value, sensory acceptance, and microbial stability of bread. *Journal of Food Measurement and Characterization*.

[55] Oyinloye P., Ajala A., Asogwa N., Galani Y. (2023). Fortification of dough with moringa, coriander, and amaranth improves the nutritional composition, health-benefiting properties, and sensory attributes of Nigerian wheat bread. *Food Science & Nutrition*.

[56] Ateeq H., Shabir Ahmad R., Imran M., Afzaal M., Asif Shah M. (2023). Valorization and structural characterization of onion peel powder for the development of functional bread. *International Journal of Food Properties*.

[57] Dossa S., Negrea M., Cocan I. (2023). Nutritional, physico-chemical, phytochemical, and rheological characteristics of composite flour substituted by baobab pulp flour (*Adansonia digitata* L.) for bread making. *Foods*.

[58] Toupal S., Copşansu S. (2023). Antioxidant and antimicrobial properties of freeze-dried banana and watermelon peel powders. *Food and Humanity*.

[59] Aboul-Enein A. M., Salama Z. A., Gaafar A. A., Aly H. F., Abou-Elella F., Ahmed H. (2016). Identification of phenolic compounds from banana peel (*Musa paradaisica* L.) as antioxidant and antimicrobial agents. *Journal of Chemical and Pharmaceutical Research*.

[60] Ragaee S., Guzar I., Dhull N., Seetharaman K. (2011). Effects of fiber addition on antioxidant capacity and nutritional quality of wheat bread. *LWT-Food Science and Technology*.

[61] Deepika, Maurya P. K. (2022). Health benefits of quercetin in age-related diseases. *Molecules*.

[62] Korus J., Witczak M., Ziobro R., Juszczak L. (2015). The influence of acorn flour on rheological properties of gluten-free dough and physical characteristics of the bread. *European Food Research and Technology*.

[63] Jahan E., Nupur A. H., Majumder S. (2023). Physico-chemical, textural and sensory properties of breads enriched with date seed powder. *Food and Humanity*.

[64] Ayoub W. S., Zahoor I., Dar A. H. (2022). Effect of incorporation of wheat bran, rice bran and banana peel powder on the mesostructure and physicochemical characteristics of biscuits. *Frontiers in Nutrition*.

[65] Shammari B. B. (2023). The influence of adding banana peel powder on the quality of pan bread. *IOP Conference Series: Earth and Environmental Science*.

[66] Khatun M., Ahmed M. W., Hossain M. M., Karmoker P., Iqbal A. (2021). Utilization of banana peel flour in biscuit making as wheat flour substitute. *European Journal of Agriculture and Food Sciences*.

[67] Shafi A., Ahmad F., Mohammad Z. H. (2022). Effect of the addition of banana peel flour on the shelf life and antioxidant properties of cookies. *ACS Food Science & Technology*.

